# Hypoxia Induced Tumor Metabolic Switch Contributes to Pancreatic Cancer Aggressiveness

**DOI:** 10.3390/cancers2042138

**Published:** 2010-12-16

**Authors:** Sophie Vasseur, Richard Tomasini, Roselyne Tournaire, Juan L. Iovanna

**Affiliations:** INSERM U624, Stress Cellulaire, Parc Scientifique et Technologique de Luminy, 163 Avenue de Luminy, BP 915¸13288 Marseille cedex 9, France; E-Mails: richard.tomasini@inserm.fr (R.T.); roselyne.tournaire@inserm.fr (R.T.); juan.iovanna@inserm.fr (J.L.I.)

**Keywords:** pancreatic cancer, hypoxia, metabolism, cachexia

## Abstract

Pancreatic ductal adenocarcinoma remains one of the most lethal of all solid tumors with an overall five-year survival rate of only 3–5%. Its aggressive biology and resistance to conventional and targeted therapeutic agents lead to a typical clinical presentation of incurable disease once diagnosed. The disease is characterized by the presence of a dense stroma of fibroblasts and inflammatory cells, termed desmoplasia, which limits the oxygen diffusion in the organ, creating a strong hypoxic environment within the tumor. In this review, we argue that hypoxia is responsible for the highly aggressive and metastatic characteristics of this tumor and drives pancreatic cancer cells to oncogenic and metabolic changes facilitating their proliferation. However, the molecular changes leading to metabolic adaptations of pancreatic cancer cells remain unclear. Cachexia is a hallmark of this disease and illustrates that this cancer is a real metabolic disease. Hence, this tumor must harbor metabolic pathways which are probably tied in a complex inter-organ dialog during the development of this cancer. Such a hypothesis would better explain how under fuel source limitation, pancreatic cancer cells are maintained, show a growth advantage, and develop metastasis.

## 1. Introduction

Pancreatic ductal adenocarcinoma (PDAC) is a poorly perfused and poorly vascularized tumor. Studies have shown that pancreatic tumors contain regions of extremely low pO2 [1]. This low intratumoral oxygen tension creates a severe hypoxic environment at the site of the tumor, which provides a strong selective pressure able to regulate tumor cell growth and to favor survival of the most aggressive malignant cells. The selected neoplastic cells exhibit extensive ability to invade to surrounding tissues with a potential to develop metastasis to distant organs even at an early stage of tumor progression.

## 2. Hypoxia: A Master Regulator of Pancreatic Cancer

**Figure 1:**
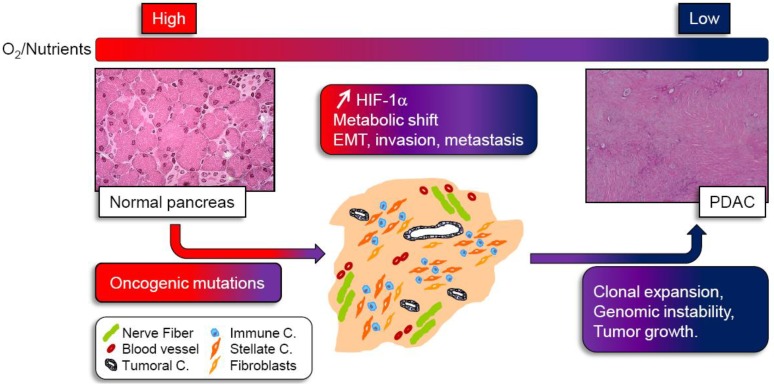
Integrating hypoxic and metabolic stress in pancreatic tumor development. Activated oncogenes and loss of tumor suppressors induce growth and proliferation of small amount of pancreatic cancer cells leading to formation of tumor mass. In this mass, the tumor cells are rapidly surrounded by a very dense stroma composed of nerve fiber, immune cells, few blood vessels, fibroblasts and stellate cells. Such desmoplasic reaction gradually reduces oxygen and nutrient supply to cancer cells. As a consequence, tumor cells activate the hypoxia-inducible factor 1α (HIF-1α) and undergo several metabolic shifts to adapt and survive. Pancreatic tumor cells harboring such changes and able to proliferate under very hypoxic and nutrient deprived conditions, promote epithelial to mesenchymal transition (EMT) and acquire a very aggressive, metastatic phenotype. Although most tumor cells experiencing metabolic stress undergo apoptosis, those surviving will be clonally expanded, and submitted to genomic instability, leading to tumor growth and pancreatic ductal adenocarcinoma formation (PDAC).

Intra-tumoral hypoxia is a major factor contributing to cancer progression, but the precise mechanisms by which hypoxic conditions may affect progression still remain poorly understood. One of the main observations that revealed the important intra-tumoral hypoxia in pancreatic cancer is the elevation of Hypoxia-inducible factor-1α (HIF-1α), the major transcription factor activated under hypoxic conditions [2,3]. Schwartz *et al*. observed nuclear staining of HIF-1α in 80% of human PDAC but only in 16% of normal pancreas. Interestingly, stroma adjacent to the pancreatic ductal carcinoma also showed HIF-1α staining in 43% of cases [4]. HIF-1α has an important but complex role in regulating molecular pathways involved in tumoral initiation or development such as angiogenesis, apoptosis, proliferation, extracellular remodeling, immunosurveillance, tissue invasion and metastasis, genomic instability and glycolysis [5]. It is thus speculated that during cancer initiation and progression, this factor greatly helps pancreatic cells to gain oncogenic properties that allow them to face and overcome the hypoxic conditions, like high proliferative capacity, invasion and metastatic potential (Figure 1). As expected, eradication of HIF-1α -active cells, the most invading and metastatic cells, impairs tumor progression and dissemination [6]. Of note, *in vitro* and *in vivo* studies have also shown that use of a novel selective HIF-1α inhibitor potentiates fractionated radiation-induced pancreatic cancer cell death, with or without combined treatment with 5-fluorouracil or gemcitabine. This observation strongly suggests that tumor and stromal HIF-1α signaling enhances innate radiation resistance of hypoxic pancreatic tumor cells. Hence, HIF-1α inhibitors appear potentially and clinically relevant enhancers of pancreatic cancer radiosensitivity [4]. Another hallmark of pancreatic cancer that is implicated in the creation of such intra-tumoral hypoxic level is the prominent desmoplastic reaction that surrounds epithelial tumor cells. In PDAC, cancer cells are “isolated and protected” by a fortress composed of activated fibroblasts (called “pancreatic stellate cells” PSCs), immune cells, extracellular matrix components and surprisingly few blood vessels [7] (Figure 1). PSCs are responsible for producing the stromal reaction in pancreatic cancer as they secrete high amounts of extracellular matrix (ECM) proteins, thereby creating a fibrotic and hypoxic environment. Such deposition of stromal proteins distorts the normal parenchymal architecture of the pancreas and limits the oxygen diffusion in the tissue. Moreover, as hypoxia increases PSCs’ activity, it maintains deposition of ECM proteins in the periacinar spaces, which exerts compression of the fine capillary network and thereby reduces oxygen diffusion. Despite being fibrogenic by the secretion of abundant type I collagen and fibronectin, PSCs harbor proangiogenic abilities through secretion of VEGF, basic fibroblast growth factor, periostin, and type I collagen [8,9,10,11]. Nonetheless, excessive ECM proteins in the periacinar spaces and perpetuation of PSCs’ activity by hypoxia overwhelm PSCs’ local proangiogenic properties, creating tissue hypoxia and pancreatic cancer [12]. The poorly vascularized architecture that imposes hypoxic stress in pancreatic cancer may help explain why antiangiogenic therapies generally fail in PDAC, and why novel therapeutic approaches targeting cancer-stroma interactions should be explored. Using mice models, Olive *et al*. showed that depletion of tumor-associated stromal tissue by inhibition of the Hedgehog (Hh) cellular signaling pathway improved gemcitabine delivery and efficacy [13]. Unexpectedly, disruption of the desmoplastic stroma through targeting of Hh increased tumor vascular density and intratumoral concentration of gemcitabine. This profound effect on tumor vasculature was associated with decreased chemoresistance and transient stabilization of the disease in treated animals. Therefore, the pancreatic tumor microenvironment appears to promote the malignant phenotype of this tumor by submitting cancer cells to high genomic instability. This phenomenon then leads to the selection of highly aggressive cells able to survive such an oxygen and nutrient deprived environment.

Along these lines, hypoxia within the neoplasic pancreatic mass is considered as an independent prognostic indicator of poor outcome. A significant risk to develop metastasis from primary cancer cells is also associated with hypoxic tumors and several explanations of how hypoxia favors increased migratory/invasive propensity of these cells are reported. The ability of hypoxic cancer cells of epithelial origin to invade surrounding tissues may involve switching on an epithelial to mesenchymal transition program (EMT), increasing their invasiveness. EMT is a complex process that regulates major events during early development and, in adult life, has a pivotal function in tissue remodeling during organ fibrosis, and cancer progression [14,15,16]. During EMT, epithelial cells lose polarization, disassemble cell–cell junctions, and switch to motile mesenchymal cells. Cells synthesize fibronectin, collagen type I, vimentin and express α-smooth muscle actin (α-SMA) or/and fibroblast-specific protein-1 (FSP-1/S100A4), acquiring a fibroblast-like phenotype. Hypoxic regulation of EMT has been shown to be involved in cancer progression and metastasis, and HIF-1α has been identified as a regulator of EMT in several cancer cell lines [17,18,19]. Exposure to hypoxia leads pancreatic tumor cells to acquire some of the accepted features of EMT such as mesenchymal markers (α-SMA or vimentin), fibroblastoid phenotype, nuclear translocation of Snail, E-cadherin down-regulation, and increased migratory/invasive propensity [20]. Moreover, early reactive oxygen species after hypoxia exposure could initiate the EMT program and a later HIF-1α-dependent release of VEGF could increase invasiveness. Thus, in the context of cancer, an EMT-like process induced by hypoxia may permit dissemination of tumor cells from the primary tumor into the surrounding stroma, setting the stage for metastatic spread. Before evading the primary tumor, hypoxic pancreatic cancer cells have to find alternative metabolic pathways that allow them to obtain energy when both oxygen and glucose are depleted. When cancer cells are under nutrient starved conditions, functional autophagic machinery appears to be an indispensable physiological reaction to sustain cell viability. However, there is probably a dual role of autophagy in cell fate decision (survival *versus* cell death) that remains a matter of controversy. Regarding pancreatic cancer, autophagy may promote cell viability in hypovascular pancreatic cancer tissue, where only limited oxygen and nutrient supplies would be expected [21].

## 3. The Metabolic Switch Still Remains Misunderstood

### 3.1. A tumoral Adaptative Metabolism

One of the major consequences of intra-tumoral hypoxia is the cell metabolic switch occurring in order to meet the requirements of tumor proliferation under low oxygen and low nutrient supply because of lack of vasculature. Since decades, it has been accepted that tumor cells display fundamental changes in pathways of energy metabolism and nutrient uptake. Otto Warburg in the 50s described for the first time that normal tissue uses glycolysis to generate around 10% of the cell’s ATP with mitochondrial oxidative phosphorylation (OXPHOS) accounting for 90% with glucose dependent ATP production. In tumor sections, however, 50% of cellular energy is produced by glycolysis with the remainder being generated in the mitochondria, even in the presence of ample oxygen to fuel mitochondrial respiration, a phenomenon known as the “Warburg effect” [22]. In clinical settings, the use of ^18^F-deoxyglucose positron emission tomography (FDG-PET) relies on this principle to image a tumor with increased glucose uptake. Analysis of tumors visualized by FDG-PET demonstrated a remarkable preponderance of metastatic cancers to trap far more glucose than normal tissue. This metabolic transformation is a consequence of the several acquired mutations that lead to cancer, and which confer a selective growth advantage and/or resistance to apoptosis to help cell survival [23]. Thus, tumor progression is driven by genetic mutations; meanwhile, environmental conditions probably provide a selective advantage that allows cells with such mutations to clonally expand (Figure 1). K-ras and the p53 tumor suppressor gene are two of the major genes mutated in pancreatic cancer and they are good examples to illustrate how nutrient or oxygen deprived conditions select particular genetic alterations. It has been demonstrated that in tumor cells, the low glucose environment favors the selection of cells with K-ras mutation [24] whereas hypoxic conditions may favor selection of cells with p53 mutation [25]. Another study by Chen *et al*. recently showed that inhibition of the mitochondrial ribosomal protein L28 (MRPL28) in a human pancreatic cancer cell line alters mitochondrial oxygen consumption, resulting in slower growth *in vitro* at normoxia, but paradoxically, an accelerated growth *in vivo* when oxygen is limited. This reduced mitochondrial function was also associated with a compensatory increase in glycolytic energy production, attesting that the cellular metabolism has changed to more closely match the Warburg observations. Hence, this study points to the concept that it is the hypoxic environment where oxygen is limiting that favors growth of cells harboring mitochondrial mutations [26]. Other authors also reported that survival of established human pancreatic cell lines was reduced when cultured with glycolytic inhibitors, and this phenomenon was correlated to lowered expression of total K-ras protein [27]. As cancer cells differ from healthy cells due to a plethora of molecular changes that are mechanistically linked to metabolic reprogramming, the metabolic signature of each type of tumor is strongly associated to oncogenic gain-of-function events or loss of tumor suppressors occurring in the tumor associated cells. One of the principal mechanisms of anaerobic (and aerobic) glycolysis resides in the activation of HIF-1α by hypoxic stress but also results in oncogenic, inflammatory, metabolic, and oxidative stress [28]. There are many genetic events involved in PDAC such as K-ras mutations, which, combined with decreased PTEN expression, amplify the downstream effector AKT [29,30]. This activation could then result in stimulation of glycolysis in pancreatic cancer cells, as it is well described in different type of tumors, through the induction of glucose transporters and glycolytic enzymes. The p53 tumor suppressor gene is mutated in >50% of PDAC cases. As p53 negatively regulates many molecular pathways which enhance glycolysis, inactivation of p53 can directly cause the Warburg phenomenon through several mechanisms during PDAC development. Hence, PDAC harbors many genetic changes which can be responsible for the metabolic switch of tumor cells from OXPHOS to glycolysis. In line with this, proteomic or genetic studies of human pancreatic tumors have revealed that glycolytic enzymes are increased in tumor samples as compared to normal tissues, and that glucose metabolism gene polymorphisms affect clinical outcome in pancreatic cancer [31,32]. It appears that single nucleotide polymorphism of some genes encoding glycolytic enzymes can be associated either with a longer overall survival (OS) in patient (probably because of a loss of function of the enzyme associated with such genetic alteration, decreased tumor progression and better response to therapy), or with a reduced OS due to higher enzyme activity and increased tumor growth.

### 3.2. Cachexia as a Consequence of Glutamine and Glucose Metabolism

**Figure 2:**
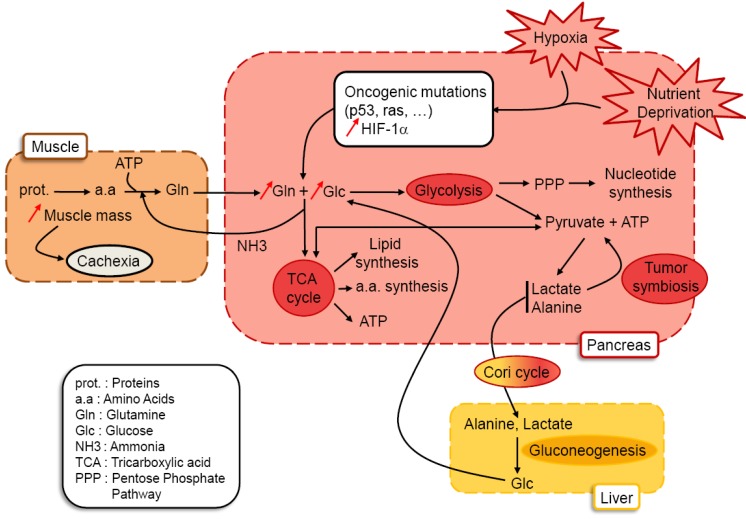
Glucose and glutamine metabolism in pancreatic tumors: hypothesis. Metabolic stress occurring in pancreatic tumor cells submitted to hypoxia and nutrient deprivation participate to selection of cancer cells harboring oncogenic mutations and activation of HIF-1α. The intimate connection between oncogenes and HIF-1α allows increased uptake of glucose and glutamine. Glucose will be processed through glycolysis to provide precursors for nucleic acids (through the pentose phosphate pathway), to produce pyruvate and ATP. Depending on oxygen availability in tumor cells, pyruvate gives either precursors for lipid and amino acid synthesis and ATP through the TCA cycle (oxidative phosphorylation) or gives lactate and alanine secreted by the tumor. Liver uptakes this pool of lactate and alanine to produce glucose used by the tumor (gluconeogenesis). Cancer cells surrounding blood vessels can also use lactate secreted by hypoxic cells as the principal substrate for mitochondrial oxidative phosphorylation (tumor symbiosis). Glutamine metabolism through the TCA cycle participates in lipid and amino acid synthesis, produces some of the pyruvate pool and ATP, giving secreted NH3 (ammonia) used by muscles to build up glutamine pools used by the tumor. Proteolysis occurring in muscles to produce glutamine is ATP consuming, occurs to the detriment of muscle mass and is responsible for cachexia.

Tumor cell metabolism is designed to support the synthesis of a complete daughter cell, every cell cycle. As growing tumor has increased metabolic demands outstripping nutrient supply; a challenge is to engage cellular metabolism changes allowing tumor cells to (1) coordinate production of the biochemical precursors necessary for macromolecular synthesis, and to (2) maintain cellular bioenergetics and integrity without impairment of cell growth, proliferation and viability. Shifting substrates from energy production to molecular synthesis could provide tumor cells with additional capacity for macromolecular production. Glycolytic pathways that produce ATP and pyruvate generate bioproducts which enter the pentose phosphate pathway (PPP) to give ribose-5-phosphate (Rib-5-P) and NADPH, keys intermediate in nucleotide biosynthesis. During glycolysis, conversion of glucose to fructose provides an essential substrate for the nonoxidative PPP. It has been demonstrated that although glucose is the major substrate for cancer cell proliferation, pancreatic cancer cells can also uptake and use fructose for growth, and to a greater extent than glucose to generate nucleic acids [33]. Another major intermediate, pyruvate, is converted to lactate by lactate dehydrogenase (LDH-A), and is readily secreted into the extracellular environment. Depending on oxygen availability in the tumor, pyruvate is redirected into the mitochondria to serve as a carbon source for amino acid and lipid synthesis [34] (Figure 2).

In addition to increased glycolysis, cancer cells also show increased use of glutamine, the most abundant amino acid in the plasma and the major carrier of nitrogen between organs. The type of oncogenes activated in the tumor cells will influence glutamine metabolism [35,36,37]. Thus, tumor genetics can dictate cellular dependence on glutamine or glucose for survival. Detection of small size early pancreatic carcinoma lesions using FDG-PET imagery appears sometimes difficult. This can be explained because they are weakly detectable due to their small size, or because these pancreatic cancer cells also use alternative metabolic pathways other than glycolysis. Then, it appears important to hypothesize that pancreatic tumors do not rely only on glucose uptake, and to point out the question: except glucose, what fuels the highly hypoxic pancreatic cancer cells? Among patients with pancreatic cancer, 85% become cachectic (atrophy of adipose tissues and skeletal muscles), the weight loss increasing to a median of 24.5% at the last assessment before death [38]. Glutamine metabolism is tied to cachexia, as more than 90% of the body’s glutamine stores are in the muscle, and glutamine is the major amino acid exported from the muscle during catabolic stress. Glutamine’s consumption by rapidly proliferating tumor cells might be the trigger for cachexia [39]. Hence, we can consider that proliferation of pancreatic cancer cells relies both on glucose and glutamine, which are converted to biomass (nucleic acids, proteins and lipids) and to by-products secreted as a result of their metabolism such as lactate, alanine and ammonia [40] (Figure 2). Moreover, it should be emphasized that lack of oxygen availability in the pancreatic tumor more than tumor genetics will significantly influence and orient cellular metabolism towards glucose or glutamine processing. Little is known about the effects of hypoxia on glutamine metabolism [41]. Presumably, glutamine metabolism is required to maintain amino acid and nucleotide synthesis needed for the cells to survive, as it was proposed for neuroblastoma cells in which hypoxia could stimulate the import of glutamine [42]. However, how hypoxia influences glutamine metabolism and whether glutamine influences cell survival during hypoxic stress are still unanswered questions. Accelerated and massive metabolism of glucose and glutamine by pancreatic tumors probably affects metabolism in the rest of the body. It was suggested that the cachectic phenotype observed in cancers consists of a combination of complex changes in muscle and liver metabolism to the benefit of the tumor, culminating in weight loss [40]. We can then speculate that in cachectic patients harboring a pancreatic tumor, there are inter-organ metabolic cycles. As mentioned before, lactate, alanine and ammonia are by-products derived from consumption of glucose and glutamine by the tumor. Secretion of lactate by hypoxic cells within tumors may be taken up by better oxygenated cells within the tumor and used as a respiratory fuel in the mitochondria, creating a symbiotic metabolism between both cells [43]. In addition, in the tumor, high levels of lactate was positively correlated with the likelihood of metastasis and tumor recurrence and negatively with patient survival [44]. Lactate and alanine are delivered to the liver and used to produce glucose through gluconeogenesis, which can then return to the tumor (the Cori cycle) (Figure 2). It has to be mentioned that the Cori cycle may account for an additional loss of energy in cancer patients of 300 kcal/day [45]. Moreover, the increased hepatic glucose production is partially due to a lack of inhibition of gluconeogenesis by insulin produced by endocrine pancreatic cells [46]. Lastly, the by-product ammonia will be either processed through the urea cycle, or will serve as a substrate for production of a new glutamine pool in the muscle during protein catabolism and glucose metabolism. Both the Cori cycle and the putative glutamine-ammonia cycle deliver energy to the tumor. However, their processing is energy consuming in the organs involved, driving up whole-body energy expenditure as is typically observed in cancer cachexia.

**Figure 3:**
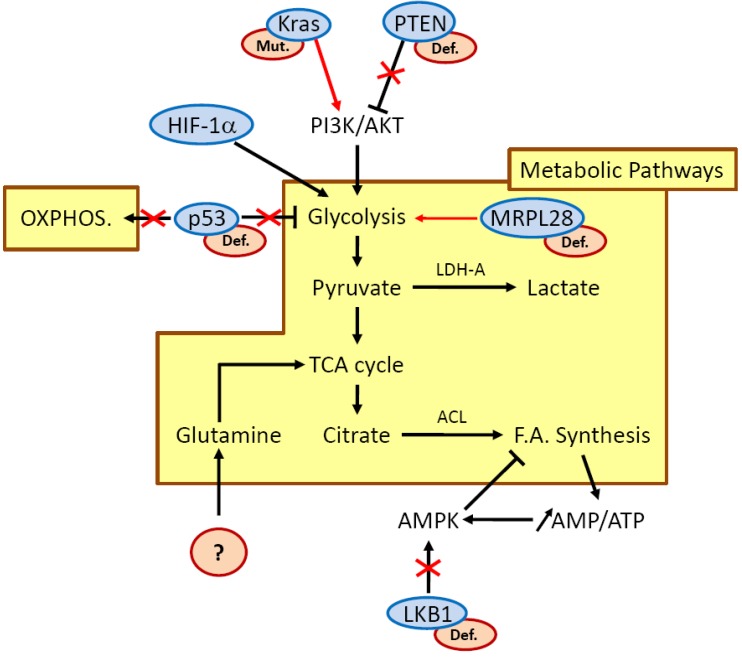
Molecular pathways involved in metabolic reprogramming in pancreatic cancer cells downstream of hypoxia and nutrient starvation. Red crosses and red arrows illustrate modulation of pathways downstream of oncogenes or tumor suppressor genes when they appear mutated (mut.) or defective (def.) (red ovals). Molecular events affecting K-ras, PTEN, p53 and mitochondrial proteins contribute to enhance glycolysis. LKB1 defect alters AMPK activation and probably impacts regulation of fatty acid (F.A.) synthesis. In PDAC, genetic alterations leading to increased glutamine uptake remain unknown. ACL, ATP citrate lyase; LDH-A, lactate dehydrogenase A; MRPL28, mitochondrial ribosomal protein L28; OXPHOS, oxidative phosphorylation; TCA, tricarboxylic acid.

Those metabolic cascades that control energy production and biosynthesis can be regulated in two ways: they are either activated by oncoproteins or, following loss of tumor suppressor genes, they appear upregulated. In a tumor, growth factors will activate the PI3K/AKT pathway to promote the anabolic energy consuming pathway, such as fatty acid synthesis. Carbon source for fatty acid synthesis is derived from glucose, which is converted to acetyl-CoA and then citrate in the TCA cycle. In highly proliferating cells, the enzyme ATP citrate lyase (ACL) uses citrate as a carbon source to generate fatty acids, and disruption of ACL impairs tumor growth [47]. Moreover, glutamine participates in lipid synthesis as it represents another carbon source to maintain citrate production in the first step of the TCA cycle. Hence, both glutamine and glucose support generation of products needed for fatty acid synthesis. However, such processes result in an increased ratio of AMP/ATP within the tumor cell, and activate the metabolic sensor AMP-activated protein kinase (AMPK) to drive catabolic energy-producing response (such as fatty acid oxidation). As pancreatic tumor cells are highly proliferative, the balance between fatty acid synthesis and oxidation must be critical and probably accounts for a major part of the increase of the tumor and of the atrophy of adipose tissues in patients with PDAC (Figure 3).

### 3.3. LKB1/AMPK Is a Potential Candidate for Treating Pancreatic Cancer Cells by Regulating Their Metabolism

The metabolic sensor AMPK is regulated by the tumor suppressor LKB1 and its activation results in a decrease in ATP-consuming processes and an increase in ATP production (Figure 3). Germ line loss-of-function mutations of LKB1 are associated with Peutz-Jeghers syndrome (PJS), a disease characterized by gastrointestinal neoplasms marked by a high risk of pancreatic cancer [48]. Around 5% of pancreatic cancers show somatic inactivating mutations in LKB1, and levels of LKB1 expression have been shown to be decreased in around 20% of human PDAC [49]. Conditional LKB1 deletion in mice pancreatic epithelium demonstrated defects in acinar cell polarity, cytoskeletal organization and loss of tight junctions concomitantly with an inactivation of the AMPK/MARK/SAD family kinases. Mice rapidly develop pancreatic serous cystadenomas, but LKB1 loss alone is not sufficient to drive formation of PDAC [50]. In LKB1 heterozygous mice, gastrointestinal polyps were associated with deregulated AMPK-TSC-mTOR signaling. Interestingly, additional mutation of the K-ras oncogene concomitantly with LKB1 deletion in lung is able to drive adenocarcinoma formation in this organ, raising the possibility that both deletion of LKB1 and constitutive activation of K-ras in pancreatic epithelium could also be responsible for adenocarcinoma development [51]. This data has recently been confirmed, as it was demonstrated using a mouse model that LKB1 haploinsufficiency cooperates with oncogenic Kras^G12D^ to cause PDAC. These tumors harbored decreased p53/p21-dependent growth arrest. Remarkably, patients with PDAC and low levels of LKB1 also present low levels of p21 [49]. Hence, there is compiling evidence that energy sensing, cell polarity and tumor progression in PDAC are integrated processes under the control of the LKB1-AMPK pathway. Despite the existence of compiling data showing that AMPK activation by energy stress enhances uptake and metabolism of glucose [52], little is known about how LKB1 influences metabolic processes in PDAC. In other organs, studies have demonstrated that somatic deletion of LKB1 in the mouse skeletal muscle produces defects in glucose uptake and loss of AMPK activation [53]. Moreover, LKB1 deletion in liver also causes metabolic defects and loss of activity of AMPK [54]. Genes associated with LKB1 loss and progression to invasive and metastatic lung tumors have been identified as well as micro-RNAs regulating LKB1/AMPK signaling which confers adaptation to metabolic stress in glioma cells [55,56]. Worldwide, the AMPK is currently activated by using metformin as a treatment for patients suffering from type 2 diabetes. Administration of metformin reduces risk of pancreatic cancer with a range of 62% in patients with type 2 diabetes mellitus [57]. However, mechanisms by which metformin lowers pancreatic cancer incidence are not clearly understood. Data showed that metformin lowers blood glucose levels through reduced hepatic gluconeogenesis and increases glucose uptake in skeletal muscles and adipose tissue [54]. More recently, it has been demonstrated that metformin could inhibit the growth of human pancreatic cancer cells xenografted in nude mice [58].

## 4. Conclusion

In summary, understanding the pathways that regulate pancreatic tumor cell metabolism may lead to a better understanding of the development and progression of this cancer. As current therapies offer very limited survival benefits, novel therapeutic strategies are urgently needed and metabolic treatment could be a new open window. Pancreatic tumor progression is driven by its genetic mutations but hypoxic and nutrient deprived environmental conditions of this tumor probably select highly aggressive mutated cells. As mutant cells exposed to hypoxia exhibit enhanced glucose and probably glutamine uptake as well as glycolysis to survive in such a deprived environment, they must harbor specific metabolic signatures allowing them to clonally expand to form the tumor. Targeting the new metabolic pathways engaged by those tumorigenic cells could limit the tumor growth. We argue here that pancreatic cancer cells are metabolically flexible and probably rely on different metabolic pathways at the same time to adapt to an environment in which glucose or oxygen, or both, are limited. Combined therapies to avoid resistance to a single anti-metabolic drug is challenging but appears to be a good strategy to counteract the tumor cell metabolism plasticity and pancreatic tumor progression.
